# Antibiotic Residues and Resistance in Three Wastewater Treatment Plants in Romania

**DOI:** 10.3390/antibiotics13080780

**Published:** 2024-08-19

**Authors:** Svetlana Iuliana Polianciuc, Alexandra Ciorîță, Maria Loredana Soran, Ildiko Lung, Béla Kiss, Maria Georgia Ștefan, Daniel Corneliu Leucuța, Anca Elena Gurzău, Rahela Carpa, Liora Mihaela Colobațiu, Felicia Loghin

**Affiliations:** 1 Department of Toxicology, Faculty of Pharmacy, Iuliu Hațieganu University of Medicine and Pharmacy, 400349 Cluj-Napoca, Romania; 2 Electon Microscopy Centre, Faculty of Biology and Geology, Babeș-Bolyai University, 400006 Cluj-Napoca, Romania; 3 Integrated Electron Microscopy Laboratory, National Institute for Research and Development of Isotopic and Molecular Technologies, 400293 Cluj-Napoca, Romania; 4 Department of Physics of Nanostructured Systems, National Institute for Research and Development of Isotopic and Molecular Technologies, 400293 Cluj-Napoca, Romania; 5 Department of Medical Informatics and Biostatistics, Iuliu Hațieganu University of Medicine and Pharmacy, 400349 Cluj-Napoca, Romania; 6 Department of Public Health, Faculty of Political, Administrative and Communication Sciences, Babeș-Bolyai University, 400095 Cluj-Napoca, Romania; 7 Department of Molecular Biology and Biotechnology, Faculty of Biology and Geology, Babeș-Bolyai University, 400015 Cluj Napoca, Romania; 8 Department of Medical Devices, Faculty of Pharmacy, Iuliu Hațieganu University of Medicine and Pharmacy, 400349 Cluj-Napoca, Romania

**Keywords:** antibiotics, antibiotic resistance, risk quotient, environment, wastewaters

## Abstract

This study evaluates antibiotic residues and bacterial loads in influent and effluent samples from three wastewater treatment plants (WWTPs) in Romania, across four seasons from 2021 to 2022. Analytical methods included solid-phase extraction and high-performance liquid chromatography (HPLC) to quantify antibiotic concentrations, while microbiological assays estimated bacterial loads and assessed antibiotic resistance patterns. Statistical analyses explored the impact of environmental factors such as temperature and rainfall on antibiotic levels. The results showed significant seasonal variations, with higher antibiotic concentrations in warmer seasons. Antibiotic removal efficiency varied among WWTPs, with some antibiotics being effectively removed and others persisting in the effluent, posing high environmental risks and potential for antibiotic resistance development. Bacterial loads were higher in spring and summer, correlating with increased temperatures. Eight bacterial strains were isolated, with higher resistance during warmer seasons, particularly to amoxicillin and clarithromycin.

## 1. Introduction

Antibiotics are essential tools in modern medicine, significantly decreasing mortality and morbidity rates from infectious diseases [1]. But, antibiotic pollution in aquatic environments has emerged as a global environmental challenge, with implications for human and ecological well-being.

The rise of antibiotic usage has led to the exposure of bacterial communities and ecosystems to a significant amount of antibiotic residues. Also, the widespread use of antibiotics has led to the emergence and spread of antibiotic-resistant bacteria (ARB), posing a severe threat to human health. Wastewater treatment plants (WWTPs) are essential in eliminating antibiotics and various contaminants from wastewater prior to its release into natural ecosystems. However, there is a substantial possibility that WWTPs could themselves become major contributors to the dispersion of antibiotic residues across various environmental compartments when the treatment process is inadequate. Even if contaminated wastewater is treated in WWTPs, a complete removal of antibiotics, ARB, and ARGs (antibiotic resistance genes) is impossible in conventional WWTPs [2,3]. WWTPs are considered major reservoirs of ARB and ARGs due to the discharge of untreated or partially treated wastewater containing antibiotic residues [4]. It is crucial to monitor antibiotic concentrations in both influents and effluents and to assess the effectiveness of the treatment processes in reducing the antibiotic contamination of wastewater. The incomplete removal of antibiotics from wastewater is a major concern because antibiotics can re-enter the environment through wastewater treatment plants and thus contaminate drinking water, soil, and waterways [5]. Antibiotic environmental contamination could increase the resistant bacterial population or maintain the selective pressure on it [6].

The analysis of influents and effluents from WWTPs could provide useful information about medication use and misuse [7]. Antibiotics are not fully metabolized in animals or humans, resulting in the excretion of 30–90% of the administered antibiotics as parent compounds through urine and feces. This introduces antibiotics into the environment, where they can persist due to their varying half-lives. For instance, the half-lives of azithromycin, amoxicillin, and ciprofloxacin are less than 5 h, less than a day, and less than 46 h, respectively, while norfloxacin persists for up to 77 days [5]. These half-life variations underscore antibiotics’ diverse temporal behavior in aquatic systems. The degradation of antibiotics in WWTPs and the environment is influenced by several interconnected processes, including seasonal variations, which can affect the fate and transport of antibiotics within WWTPs. Therefore, a thorough investigation into the impact of the changing seasons on antibiotic removal and environmental risk is essential for optimizing treatment processes. [5]. The release of antibiotics into the environment, coupled with their persistence and potential to generate environmentally concerning metabolites, necessitates concerted efforts to regulate and control these contaminants.

This study focuses on evaluating the presence of the following antibiotics, amoxicillin, piperacillin, ciprofloxacin, norfloxacin, azithromycin, clarithromycin, and doxycycline, in three Romanian WWTPs, over the four seasons in the years of 2021–2022. These antibiotics were chosen due to their widespread use in human medicine, veterinary medicine, and animal farms, leading to their frequent detection in wastewater treatment plants and the environment. These antibiotics were selected through inquiries made to hospital pharmacies with high antibiotic consumption, such as those in infectious disease and pneumology hospitals, as well as community pharmacies. These inquiries focused on the most frequently dispensed antibiotics during 2020 and 2021, covering the COVID-19 pandemic period, which saw an increase in antibiotic usage in Romania compared to average European consumption. National reports from the ECDC further confirm this increased usage [8]. Also, some of these antibiotics, including amoxicillin, ciprofloxacin, azithromycin, and clarithromycin, are listed on the environmental watch list due to their potential as aquatic pollutants, further justifying their inclusion in this study [9].

For the first time, the central-western region of Romania is considered for analyzing antibiotics within this study in the influents and effluents of three WWTPs which are discharging their effluents into the same river. Our objectives were to quantify the selected antibiotics in influent and effluent samples, assess the removal efficiency of the WWTPs, and evaluate the risk posed by the identified residues to aquatic ecosystems. Additionally, we aimed to understand the seasonal variation in antibiotic concentrations and their correlation with the development of antibiotic resistance in bacterial populations. A preliminary microbiological assay was conducted as an initial step in this study to lay the groundwork for the planned advanced molecular analyses. The comprehensive approach of the present study combines chemical analysis, microbiological assays, and environmental risk assessment to provide an understanding of the challenges posed by antibiotic residues in wastewater.

## 2. Results

### 2.1. Occurrence and Seasonal Variation of Antibiotics in Wastewaters

The present study measured the residual antibiotic concentrations that occurred in the influents and effluents of three WWTPs across four seasons. Table 1 shows that only one antibiotic, amoxicillin, displayed a statistically significant concentration difference between the influent and effluent samples. The mean difference in concentration for amoxicillin was 7.11 µg/mL, with a 95% confidence interval ranging from 2.84 to 19.98 µg/mL. Therefore, despite the confidence interval suggesting a potential difference in concentration, the *p*-value of 0.093 indicates that this result is not statistically significant. Also, the remaining six antibiotics (AZT, CIP, CLT, DOX, PIP, and NOR) did not exhibit statistically significant differences between influent and effluent concentrations.

Regarding the seasonal variations in antibiotic concentrations, most of the *p*-values (Table 2) are less than 0.05, indicating statistically significant results. Differences in seasonal antibiotic concentrations were observed for both influent and effluent samples. In the spring and summer, high concentrations of amoxicillin were detected in the influents of all WWTPs, with particularly high effluent levels observed in WWTP C (31.177 µg/mL) and WWTP B (31.163 µg/mL). Piperacillin showed extremely high concentrations in the effluent of WWTP B during autumn (35.056 µg/mL), while ciprofloxacin and norfloxacin were generally low or undetectable, except for notable influent levels in WWTP B during spring (9.213 µg/mL and 16.578 µg/mL, respectively). Azithromycin and clarithromycin were largely undetected, except for trace levels in specific influents, and doxycycline exhibited higher effluent levels in autumn and winter in WWTP C and WWTP B.

**Figure 1:**
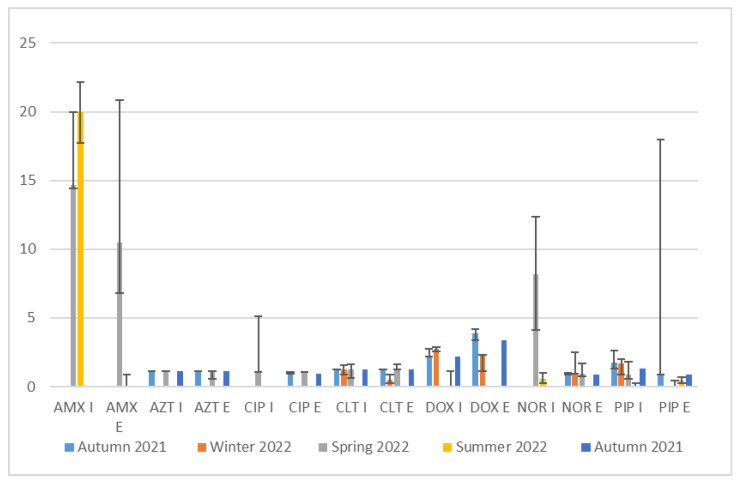
The median variability in antibiotic concentrations across different seasons and sampling points.

Figure 1 includes error bars representing the median variability in antibiotic concentrations across different seasons and sampling points. The lower error bars indicate the first quartile (Q1), and the upper error bars indicate the median value’s third quartile (Q3). Significant variability was observed for amoxicillin (Q3 median values reaching up to 22.16 μg/mL) in both influents and effluents (Q3 median values reaching up to 20.82 μg/mL), particularly during the summer of 2022 and the autumn of 2021, highlighting fluctuating concentrations. Azithromycin and ciprofloxacin showed consistent low concentrations and high variability in specific seasons. Clarithromycin and doxycycline exhibited moderate to high variability, indicating inconsistent detection and removal efficiency across seasons. Norfloxacin and piperacillin also displayed significant variability, with influent concentrations peaking in the spring of 2022 and the autumn of 2021.

### 2.2. Antibiotic Removal Efficiency from Wastewater Treatment Plants

Antibiotic removal efficiency (RE%) was calculated based on the equation described by Douziech et al. [10],

RE = (1 − exp (LN(CEWW/CIWW)) × 100%) CEWW

CIWW—Influent antibiotic concentration
CEWW—Effluent antibiotic concentration

Table 3 shows the variations of the antibiotic removal rates during four seasons for the three WWTPs, which varied between 3% and −315%. Attempts to establish statistical significance in the correlation between wastewater treatment efficacy and antibiotic levels yielded no significant results (Table 4). Piperacillin had very high removal rates in all seasons except for the autumn season in WWTP C, where a low negative removal rate was obtained. Norfloxacin had removal rates that varied between 70% and 100%. Clarithromycin (3% and 100%) and doxycycline (19% and −105%) had a large range of removal rates, which could be explained by the fact that they are persistent antibiotics and have the capacity of being adsorbed by solid matrices such as sludge. We noticed negative antibiotic removal efficiency for amoxicillin (−23% in WWTP B in Q2), for doxycycline (−30% in WWTP A in Q4, −105% in WWTP B in Q4, and −20% in WWTP C in Q4) and piperacillin (−315% in WWTP C in Q4).

### 2.3. Antibiotic Resistance Profiles of Isolated Bacteria

For samples where CFU were observed (Table 5), the isolation of at least one bacterial strain was conducted. There were a total of 24 isolates, out of which 8 could be further investigated by Gram coloration, antibiograms, and SEM.

### 2.4. Gram Staining

The eight isolates (six isolates from the spring season and two from the summer season) were tested to determine their Gram group (Table 6). Out of these, five strains were Gram-negative (most probably Gram-negative coliforms), one strain was Gram-positive, and two were inconclusive.

## 3. Discussions

### 3.1. Comparison of Antibiotic Concentrations with Other Studies

The data from our study reflect the trends observed in other studies, confirming the presence of these antibiotics in urban wastewater systems [11,12,13,14,15]. In the study by Mirzaei et al. [16], antibiotic concentrations in WWTPs were found to vary seasonally, with our findings indicating higher concentrations detected in the warmer months compared to the winter season. A study by Chukwu et al. [17] reported mean concentrations of antibiotics such as sulfamethoxazole reaching up to 0.28618 μg/mL in WWTP effluent, indicating substantial pollution levels. Also, seasonal variations in antibiotic concentrations were observed, with influent levels reaching several µg/mL. A study by Faleye et al. [18] reported influent antibiotic concentrations ranging from 1.3 ng/L of azithromycin to 81,748 ng/L of ciprofloxacin. A review by Madikizela et al. [19] reported pharmaceutical concentrations in the range of ng/L to µg/L in African water bodies. A comprehensive study conducted across Europe has reported various antibiotics such as ciprofloxacin, azithromycin, and clarithromycin in WWTP effluents with concentrations reaching up to several µg/mL, although these were lower than the levels observed in our study [20]. However, it is important to note that most European studies reported antibiotic levels in WWTP effluents that were lower than those observed in our study. This discrepancy could be attributed to regional differences in antibiotic usage and disposal practices, as well as variations in the efficiency of wastewater treatment processes. Our results align with other studies, which indicate significant pollution levels in pharmaceutical effluents [21].

### 3.2. Variability of Antibiotic Removal Efficiencies

The statistical mean and median data (Table 4) for antibiotic removal efficiency for three WWTPs during four seasons confirm that the WWTPs’ methods were not highly effective in removing most antibiotics from the wastewater. Also, the antibiotic removal efficiencies of the WWTPs were highly variable, depending on the specific antibiotic. The removal efficiency was very high for some antibiotics, such as amoxicillin, norfloxacin, and piperacillin, with mean and median values above 95%. Although good removal efficiency by treatment processes was observed for several antibiotics, most antibiotics were still present in WWTP effluents. The removal levels, such as azithromycin, ciprofloxacin, doxycycline, and clarithromycin, were much lower, with mean removal efficiencies of 20% or less.

The negative antibiotic removal for doxycycline and piperacillin during the autumn season (Q4) can be attributed to higher organic loads and runoff, which overload microbial communities and reduce their efficiency. Increased amounts of suspended solids lead to the adsorption of antibiotics onto particles, making them less available for degradation. Lower temperatures in autumn further slow microbial activity. These factors collectively reduce antibiotic removal efficiency, whereas other seasons show removal rates between 19% and 100%. Amoxicillin showed lower or negative removal rates for the spring season, but the removal rates were higher for the summer season, from 93% to 100%.

Similar results to those obtained by the present study were reported by other research groups [3,22,23,24,25]. Zheng et al. noticed the highest antibiotic removal rate during the summer season [26]. Also, Mozaz et al. [20] mentioned that better antibiotic removal rates were obtained for temperatures of 15–20 °C than for those below 10 °C. Higher antibiotic removal rates during the summer season can be attributed to elevated temperatures, which enhance the activity of microorganisms responsible for degrading antibiotics in wastewater treatment processes. Warmer temperatures improve the metabolic rates of these microorganisms, facilitating a more efficient breakdown of antibiotic compounds.

Several factors contribute to the overall low effectiveness of antibiotic removal in WWTPs. Firstly, processes like dilution, degradation, and various treatment methods (physical, chemical, and biological) affect antibiotic removal efficiency. Higher antibiotic levels in effluents than in influents may result from the cleavage of phase II metabolites (glucuronides and sulfates) during treatment, releasing parent compounds. Secondly, certain antibiotics can be metabolized or transformed by microorganisms, reducing their concentration or removing them completely. Antibiotics can also be trapped within biofilms on WWTP surfaces, protecting them from degradation and allowing accumulation. Thirdly, adsorption–desorption processes can cause antibiotics to adhere to solid particles like sludge, extracting them from the liquid phase. Lastly, many WWTPs are not specifically designed for antibiotic removal, making this a particularly complex challenge [5,12,16,27,28]. Although they have the potential to degrade, antibiotics are frequently detected in the environment, placing them in the category of pseudo-persistent pollutants. A study of the effluents from 90 WWTPs in the European Union revealed a high detection frequency for antibiotics such as trimethoprim, ciprofloxacin, and sulfamethoxazole, with rates of 93%, 90%, and 83%, respectively [5].

### 3.3. Factors Influencing the Variation of Antibiotic Concentrations

The fluctuation in antibiotic concentrations across different seasons can be attributed to various factors, including antibiotic usage patterns, seasonal consumption spikes, pandemic-related increases, the population served, anthropogenic activities, agricultural discharges, inadequate environmental regulations, poor pharmaceutical waste management, increased amounts of travel, and higher illness susceptibility [29]. In 2021–2022, Romania had the highest antibiotic consumption in Europe at 25.9 DDD per 1000 inhabitants per day, surpassing the EU average of 19.4 DDD. Contributing factors include over-the-counter availability and socio-economic barriers. Inappropriate disposal of antibiotics further exacerbates environmental contamination. Romania’s pharmaceutical waste management needs urgent reform to meet European standards [30,31].

It is important to note that the differences in concentrations of antibiotics in wastewater can vary widely, even within the same season, and can be caused by fluctuations in antibiotic usage levels [32]. The error bars (Figure 1) explain the seasonal variability of antibiotic concentrations in the influents and effluents of WWTPs. High variability suggests fluctuating usage patterns or inconsistent removal efficiencies, while low variability indicates more stable conditions. Therefore, these observed patterns are essential for assessing the impact of wastewater treatment on antibiotic levels. Also, the analysis emphasizes the need for continuous monitoring and adaptive management in WWTP operations to address the challenges posed by antibiotic residues.

A limitation of the study is that single seasonal sampling may not capture the full range of antibiotic concentrations due to potential fluctuations in consumption. Increased sampling frequency or real-time monitoring could provide a more accurate understanding of the relationship between antibiotic consumption and wastewater concentrations [33]. Similar trends have been observed regarding the correlation between the fluctuation in antibiotic concentrations and the prevalence of bacterial infections, as noted in various studies [20,34]. This correlation suggests that as antibiotic levels vary, there is a corresponding change in the incidence of bacterial infections. Such findings underscore the interconnected relationship between antibiotic use and the dynamics of bacterial infection rates [1,35].

#### 3.3.1. The Influence of Air Temperature and Rainfall on Antibiotic Concentration Variability

Statistical analysis was performed to explore the possible associations between antibiotic concentrations and the mean monthly temperature or the mean monthly rainfall. Statistically significant associations were found between the mean monthly temperature or the mean monthly rainfall and amoxicillin and doxycycline concentrations, both in univariate and multivariate models (Table 7, Table 8, Table 9 and Table 10). For both independent variables, higher values are associated with increased concentrations of amoxicillin but with decreased concentrations of doxycycline. The models, including mean monthly temperatures, had the highest determination coefficients, indicating an important effect of this variable on antibiotic concentrations. The determination coefficients were higher in the models predicting doxycycline concentrations than in those predicting amoxicillin. The treatment process of wastewater was not associated in a statistically significant manner with the antibiotic concentrations. No associations with the temperature and rainfall were noticed for the other antibiotics.

The model with mean monthly temperature as an independent variable shows a positive correlation with amoxicillin levels and has a higher determination coefficient, suggesting a predictive power of temperature on amoxicillin concentration. This might indicate that temperature increases lead to conditions that either promote persistence or reduce the degradation of amoxicillin in the environment. Rainfall also shows a significant positive effect on amoxicillin concentrations but with a lower determination coefficient than temperature. This might suggest that while rainfall impacts amoxicillin levels, it does so less consistently or predictably than temperature.

For doxycycline, the negative coefficient with temperature indicates an inverse relationship, where higher temperatures might enhance the degradation or reduce the persistence of doxycycline in wastewater. The high determination coefficient in these models highlights temperature as a critical factor affecting doxycycline levels. Therefore, temperature has a more consistent and predictable influence on the degradation and persistence of antibiotics in wastewater compared to rainfall, which can vary in its effects.

These results indicate that these environmental factors could play a role in the variability of antibiotic concentrations in wastewater, specifically for amoxicillin and doxycycline. Both antibiotics exhibit different stability profiles in wastewater. This difference could be due to the chemical properties of the antibiotics, such as their solubility and stability under varying environmental conditions. For instance, a study on the ecological degradation of doxycycline demonstrated that temperature and H₂O₂ concentration significantly affect its residual concentration [36]. Similar to temperature, higher rainfall correlates with lower doxycycline concentrations. This could be due to dilution effects or enhanced microbial degradation stimulated by increased water content. Enhanced degradation of some antibiotics, leading to lower concentrations in the final effluent because of higher temperatures and increased rainfall, was reported in the literature [11,37].

Also, in the present study, higher concentrations of norfloxacin during the spring season (Q2) in WWTP C were observed, coinciding with high rainfall compared to other seasons and WWTPs, but without statistical significance. This suggests a potential influence of seasonal rainfall on the mobility and persistence of this antibiotic in aquatic environments. Effluents could show higher antibiotic concentrations due to inefficient wastewater treatment, while influents have reduced concentrations due to dilution. Additionally, seasonal variations in wastewater flow can influence the removal efficiency of ciprofloxacin and norfloxacin in WWTPs, with higher concentrations observed during periods of high rainfall [11].

Understanding these dynamics is crucial for optimizing wastewater treatment processes. For instance, treatments can be designed to take advantage of higher temperatures for more efficient degradation during warmer seasons and to manage the influx of antibiotics due to rainfall. Temperature plays a critical role in chemical reactions and biological treatment processes in wastewater plants, and there is a growing scientific recommendation for proactive wastewater temperature management, which would be essential for efficient treatment [38,39,40,41].

#### 3.3.2. Influence of pH and Physicochemical Characteristics on Antibiotic Removal

During autumn, the high organic load in WWTP B correlates with poor antibiotic removal efficiency due to the potential overloading of microbial communities, competition for degradation pathways, and adsorption onto particulate matter. WWTP B shows COD, chemical oxygen demand, to be at 228.16 mg/L, BOD, biochemical oxygen demand, to be at 122 mg/L, suspended solids at 170 mg/L, fixed residues at 792 mg/L, ammonia at 181 mg/L, nitrites at 31.7 mg/L, phosphorus at 84.81 mg/L, and sulfates at 64.04 mg/L, indicating significant organic and nutrient loads that lead to low or negative antibiotic removal rates. In contrast, WWTPs A and C have lower CODs (181.9 mg/L and 259 mg/L, respectively) and BODs (95.35 mg/L and 85 mg/L, respectively), suggesting better performance but still showing variable antibiotic removal rates. Additionally, WWTP A’s fixed residue is 417 mg/L, and WWTP C’s is 740 mg/L, with lower ammonia (40.88 mg/L and 45.32 mg/L, respectively) and nitrite levels (0.208 mg/L in WWTP A). Despite this, high levels of fixed residues and moderate levels of suspended solids contribute to the observed variability in antibiotic removal rates. 

WWTP A, with the largest capacity of 115,000 cbm/24 h serving 300,000 inhabitants, generally performs well but requires optimization in solid matter handling and adaptation to seasonal variations. It faces challenges with high levels of fixed residues (417 mg/L) and moderate levels of suspended solids. WWTP C, with a capacity of 4772 cbm/24 h serving 31,000 inhabitants, shows reasonable performance but struggles with high solid matter and requires improved sludge management. It also deals with high levels of fixed residues (740 mg/L) and moderate levels of suspended solids. Both plants exhibit variable antibiotic removal efficiency, typically higher in warmer months. WWTP B, with a capacity of 5673 cbm/24 h serving 19,000 inhabitants, faces significant challenges due to high organic and nutrient loads. It has poor antibiotic removal efficiency, especially in autumn (Q4), with negative rates for doxycycline (−20% in Q4) and piperacillin (−315% in Q4). All three plants share issues with high levels of fixed residues and moderate levels of suspended solids, and WWTP C requires advanced treatment technologies and seasonal adjustments to improve its performance. In our case, the high amounts of turbidity and sediment in the water samples further challenge the extraction. This interaction between the antibiotics’ inherent properties and the treatment environment elucidates why ciprofloxacin and norfloxacin might persist through or even appear in higher concentrations after wastewater treatment [42].

**Figure 2:**
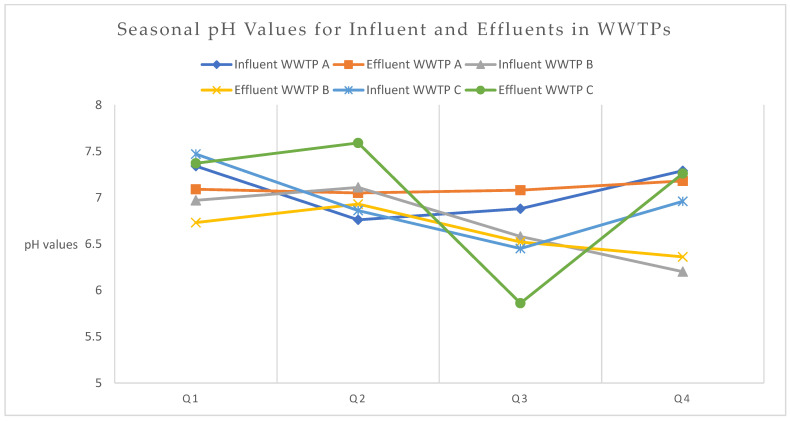
Seasonal variations in pH values in WWTPs. WWTP: Wastewater treatment plant, Q1—Winter, Q2—Spring, Q3—Summer, Q4—Autumn.

**Figure 3:**
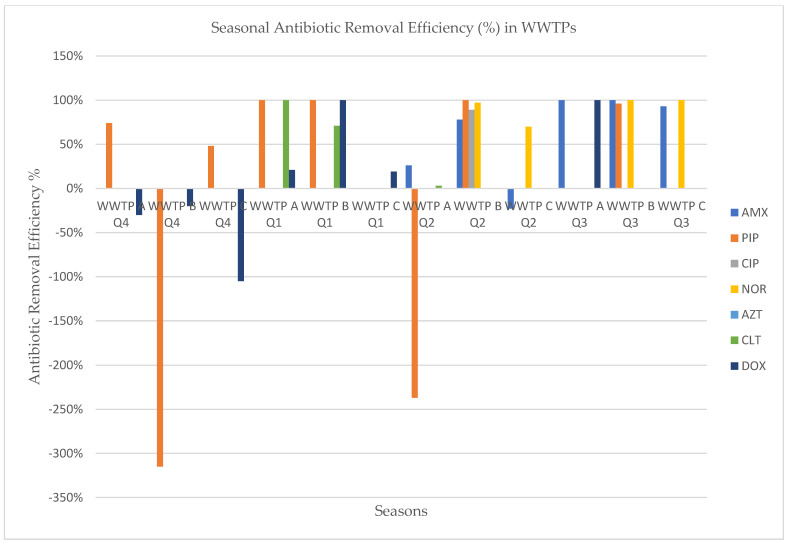
Seasonal variations in antibiotic removal efficiency in WWTPs. AMX—amoxicillin, AZT—azithromycin, CIP—ciprofloxacin, CLT—clarithromycin, DOX—doxycycline, NOR—norfloxacin, PIP—piperacillin, WWTP: Wastewater treatment plant, Q1—Winter, Q2—Spring, Q3—Summer, Q4—Autumn.

The seasonal pH values and antibiotic removal efficiencies in three WWTPs (Figure 2 and Figure 3) show the essential role of maintaining stable pH levels for effective treatment. WWTP A, with relatively stable and neutral pH values (influent: 6.76–7.34, effluent: 7.05–7.18), shows high removal efficiencies for amoxicillin and clarithromycin, particularly in Q3, while experiencing lower efficiencies and negative removal for doxycycline in Q2 and Q4. WWTP B maintains slightly consistent pH levels (influent: 6.20–7.11, effluent: 6.36–6.93), correlating with a high removal of norfloxacin in Q2 and Q3, but a negative removal for doxycycline in Q4. WWTP C, which exhibits pH fluctuations (influent: 6.45–7.47, effluent: 5.86–7.59), achieves a high removal of several antibiotics in Q2 and Q3, but poor removal efficiency for piperacillin and doxycycline in Q4 due to the low effluent pH of 5.86. These findings highlight the importance of stable, neutral pH levels in optimizing the microbial degradation of antibiotics, as significant pH fluctuations and consistently low pH conditions can reduce treatment efficiency [43,44].

For example, at a low pH, doxycycline becomes more stable and less bioavailable for microbial degradation. This could lead to its persistence in the effluent, as observed in Q4 for WWTP B. This can explain why the removal efficiency for antibiotics such as doxycycline is poor under acidic conditions in WWTP C. Regarding the effectiveness of the WWTPs, WWTP A appears to be the most effective in maintaining conditions conducive to antibiotic removal, except for DOX. WWTP B shows potential for good removal efficiency but with a slight decline in pH values across the seasons. In WWTP C, during Q4, a high organic load and a low pH correlates with poor antibiotic removal and underscores the need for better pH and physicochemical property management.

Different wastewater treatment techniques vary in their ability to remove antibiotics, with some being more effective than others. Methods such as biological processes, membrane technologies, and advanced treatments like ozonation or activated carbon adsorption can notably decrease antibiotic concentrations. However, conventional treatment methods, like those used in the WWTPs examined in this study, might not achieve the same level of effectiveness [24,45,46,47]. Also, there are several ways to improve the removal of antibiotics from wastewater. One way is to upgrade WWTPs with new technologies with advanced treatments that are designed to remove antibiotics. Also, recent studies confirm that high-rate algae ponds (HRAPs) are effective in removing antibiotics from wastewater and have potential for integration into real wastewater treatment plants [48]. Another way is to reduce the amount of antibiotics that are discharged into WWTPs. This can be achieved by educating the public about the proper use of antibiotics and by developing alternatives to antibiotics, such as bacteriophages [24,45,49,50].

### 3.4. Impact of Antibiotic Residues in Effluents of WWTPs on the Aquatic Environment

The antibiotic concentrations in the effluents were used for the assessment of their impact on the aquatic environment as it was assumed that effluents are discharged into river waters. The environmental risk associated with the occurrence of antibiotic residues in wastewaters was assessed using the risk quotient (RQ).

All the targeted antibiotics at some point showed a high level of risk based on the ecological risk assessment (RQ > 1), as can be observed in Table 11. In the Q4 season, AMX showed high risk in WWTP C, but medium risk in WWTPs A and B, even though AMX showed high concentrations in the effluents. This could be explained by the high value of the PNEC for amoxicillin (Table 11). The PNEC represents the concentration below which no adverse effects are expected; a high PNEC is indicating that the organism or endpoint has a relatively high tolerance to the antibiotic [51]. We observed the opposite effect for antibiotics like NOR, CIP, and CLT, which were detected at lower concentrations than AMX, but showed low PNEC values, which led to remarkably high RQs. As can be observed, most of the selected antibiotics showed high RQ levels; therefore, antibiotic release into the environment poses both ecological and human health risks and requires more constant biological monitoring through ecological and antibiotic resistance risk assessments.

For the protection of environmental and public health, it is crucial to evaluate the selection pressure caused by antibiotic pollution in various scenarios. The extent and magnitude of the impact at the discharge point are influenced by factors such as water depth, currents, and sediment characteristics [52]. Adopting a precautionary approach that considers dilution in the aquatic environment, this study emphasizes the need for efforts to eliminate antibiotic residues during the wastewater treatment process.

### 3.5. Analytical Limitations and Implications

In this study, a significant portion of measurable concentrations of samples fell below the LOQ. Differences below the quantification limit could be partially attributed to analytical error, hindering the accurate quantification of the actual removal efficiency. Therefore, it is difficult to definitively conclude whether observed increases in antibiotic concentration in the effluent solely reflect inefficient treatment or involve contributions from other factors like accumulation in sludge. Also, the inability of the current method to monitor the complete range relevant for environmental risk assessments—specifically, levels above PNEC-ENVs and PNEC-MICs—means that potentially harmful concentrations are often not detected and falsely reported as absent. This limitation impacts the accuracy of assessing environmental risks associated with these antibiotics.

### 3.6. Seasonal Variations in Estimated Bacterial Loads

Table 5 presents the logarithm of CFU/mL values across the four seasons for the influents and effluents of WWTPs A, B, and C. Different WWTPs show significant variations in bacterial loads both in influents and effluents.

Higher bacterial counts were observed in warmer months (spring and summer), with the highest values recorded in spring (Q2) for both the WWTP B influent (9.230 log CFU/mL) and the WWTP A influent (8.857 log CFU/mL). This increase is likely due to higher temperatures (15.5–15.4 °C in Q2 and 21.3–21.6 °C in Q3) which enhance bacterial growth, consistent with the findings of López et al. [53], who reported similar seasonal variations with bacterial concentrations reaching up to 10^8^ CFU/100 mL during warmer periods. In contrast, winter and autumn showed lower bacterial loads (3.6–4.2 °C in Q4 and 0.8–1.8 °C in Q1), likely due to inhibited bacterial activity at lower temperatures [54]. Effluent bacterial loads were generally lower, demonstrating the partial effectiveness of wastewater treatment processes. However, exceptions were noted in Q1 for WWTP A, and Q4 for WWTPs B and C, where effluent levels were similar to or slightly higher than influent levels, indicating variable treatment efficacy.

#### 3.6.1. SEM Analysis

**Figure 4:**
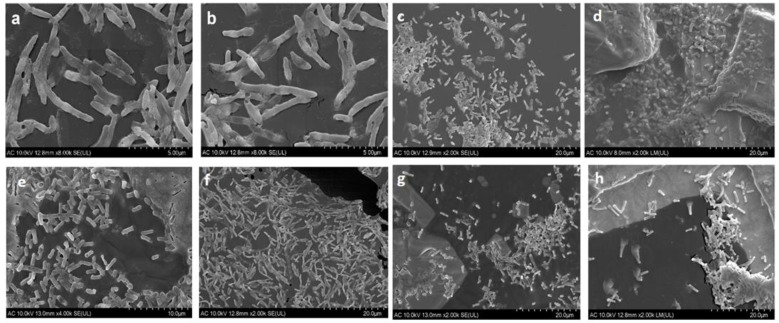
SEM micrographs of the eight isolated samples: (**a**) Q2 IWWTP A (**b**) Q2 EWWTP A (**c**) Q4 EWWTP A (**d**) Q2 EWWTP C (**e**) Q4 EWWTP C (**f**) Q2 IWWTP C, (**g**) Q3 IWWTP B, (**h**) Q2 IWWTP B. I WWTP- influent wastewater treatment plant, EWWTP—effluent wastewater treatment plant.

The SEM micrographs (Figure 4) show the bacterial morphologies and densities from various WWTPs across different seasons. Some effluent sample micrographs (Figure 4d,e) display clusters of cells, suggesting biofilm formation, which can protect bacteria from treatment processes and promote the spread of antibiotic resistance. Biofilm formation is a well-known survival strategy that protects from physical stresses and antimicrobial agents, both of which are common in WWTPs and can also facilitate horizontal gene transfer among bacteria, potentially spreading resistance traits [55,56].

Overall, the SEM analysis highlights the need to consider both seasonal variations and biofilm formation when assessing the efficiency of wastewater treatment processes. These detailed morphological observations complement the quantitative CFU data, providing a potential understanding of microbial dynamics in WWTPs.

#### 3.6.2. Analysis of Antibiotic Sensitivity Patterns

After characterization, bacterial isolates were assessed for sensitivity to seven antibiotics (Table 12). The most bacterial isolates during warmer months (Q2 and Q3) showed higher resistance to at least one antibiotic compared to those from colder months. Most isolates were generally sensitive to the antibiotics tested, but notable resistance was observed for amoxicillin and clarithromycin. Resistance to norfloxacin and ciprofloxacin varied, with some samples showing resistance or intermediate resistance. This is significant, as fluoroquinolone resistance is a critical public health issue [57]. These findings suggest that warmer conditions may facilitate the proliferation of antibiotic-resistant bacteria, potentially due to the presence of these antibiotics in the influent.

The data indicates increased resistance during the warmer seasons (Q2, Q3), likely due to factors such as higher bacterial growth rates at elevated temperatures, which can accelerate mutation rates and the spread of resistance genes [58].

The presence of antibiotic-resistant bacteria in treated effluents suggests the need to review and potentially enhance the treatment processes to better address bacterial resistance [59]. This initial microbiological assay was carried out with a narrow focus, laying the groundwork for more extensive future research. Furthermore, the SEM analysis and antibiotic sensitivity patterns provided additional support for microbiological research, particularly in the evaluation of CFU counts. These findings have also identified crucial areas for our future research on antibiotic-resistant bacteria and resistance genes.

Regular monitoring of antibiotic resistance in WWTPs is crucial for the early detection and management of resistant bacterial populations. Implementing robust antimicrobial stewardship and surveillance programs in WWTPs could help manage the risk of spreading antibiotic resistance [20,60]. The effluents from WWTPs often discharge into natural water bodies; thus, the presence of antibiotic-resistant bacteria could pose risks to ecosystems and human health. Strategies to mitigate these risks include improving disinfection processes and public health policies focusing on reducing antibiotic usage [53].

In the same sampling season, high RQs of antibiotics were observed, and resistant or intermediate-resistant strains were identified. We identified resistant or intermediate-resistant strains to all monitored antibiotics in WWTP C effluents from the Q2 spring season and high RQs for amoxicillin and norfloxacin. Also, for the Q2 season, strains resistant to amoxicillin, clarithromycin, and azithromycin were isolated in WWTP A effluents and high RQs were determined for amoxicillin, norfloxacin, and clarithromycin. There are several studies which analyzed the seasonal variation of the interrelation of antibiotics and bacteria in WWTPs [61]. For example, Shen et al. found a positive correlation between bacteria, antibiotics, and antibiotic resistance in summer due to higher microbial activity and a negative correlation in winter due to reduced microbial activity and lower temperatures [62]. Another study by Rizzo et al. found that the levels of antibiotic resistance genes in wastewater effluent were highest during the summer months. The authors suggested that this was caused by the higher concentrations of antibiotics in wastewater effluent during the summer months, which can promote the development and spread of antibiotic resistance [12].

However, it is important to note that long-term exposure, even to low concentrations of antibiotics, can also facilitate the development and dissemination of antibiotic-resistant bacteria and antibiotic resistance genes. During prolonged exposure, antibiotics exert selective pressure, thereby stimulating bacterial metabolism and the proliferation of bacteria, which can adapt to antibiotic pressure through gene mutations or horizontal gene transfer. The continuous exposure to low concentrations of antibiotics, known as sub-minimal inhibitory concentration (sub-MIC), is believed to drive the development of antimicrobial resistance in environmental microbiota. However, the relationship between antibiotic exposure and resistance selection in environmental bacterial communities is still not well understood and requires further investigation [63,64,65,66,67,68].

## 4. Materials and Methods

### 4.1. Location and Collection of Samples

**Figure 5:**
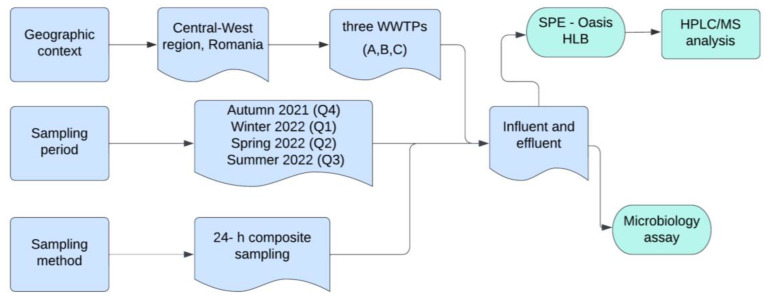
Workflow for sampling and analysis in WWTPs.

A total of twenty-four influent and effluent wastewater composite samples were seasonally collected from autumn (Q4) of 2021 to summer (Q3) of 2022 at three different WWTPs (A, B, C) located in Central-western region of Romania (Figure 5). The selection of these WWTPs was strategic, as all discharge their effluents into the same river.

Samples were collected using continuous 24 h composite sampling methods to ensure the representativeness of daily variations and were stored in sterile polyethylene bottles. Upon collection, samples were immediately chilled to 4 °C to prevent microbial activity and chemical degradation and transported promptly to the laboratory, where they were processed within 24–48 h to minimize changes in antibiotic concentrations. This required the processing of both water and sediment components for each sample, which could have contributed to the observed variability in antibiotic concentrations, as sediment fractions likely contained different levels of antibiotics. Thus, throughout this study, these samples will be referred to as ‘water samples’. It is important to note their composite nature and the potential influence of sediment on antibiotic concentrations.

### 4.2. Description of WWTP and Physicochemical Measurements

The selected WWTPs are used to treat wastewater originating from households, hospital areas, agricultural runoff, and rural areas connected to a public sewerage network. WWTP A is designed to process around 115,000 cubic meters (cbm) of wastewater/24 h, from an average of 300,000 inhabitants. WWTP B is currently treating around 4772 cbm/24 h, from around 19,000 inhabitants, while WWTP C processes around 5673 cbm/24 h, from an average 31,000 inhabitants. Specifically, WWTP A collects wastewater from many hospitals, including general, specialized, and referral hospitals. The key difference among the three WWTPs is that WWTP A manages a substantially larger volume of wastewater than the other two plants.

Table 13 presents a detailed comparative description of the treatment stages employed in three different WWTPs: WWTP A, WWTP B, and WWTP C. In the selected WWTPs, sewage treatment is performed using conventional procedures, including mechanical pre-treatment, biological, sludge, and tertiary treatment stages. 

pH measurement: The samples’ pHs (Table 14) were measured using a calibrated digital pH meter: Sension+ PH3 Basic laboratory pH and ORP Meter. Prior to measurement, the pH meter was calibrated with standard buffer solutions at pHs of 4.0, 7.0, and 10.0. All measurements were conducted at room temperature. The measurements for total nitrogen, nitrites, nitrates, ammonium, biochemical oxygen demand (BOD), chemical oxygen demand (COD), and suspended solids were provided just for the autumn season by the laboratories of the respective WWTPs. These labs followed standard operating procedures to ensure accuracy and reliability.

### 4.3. Air Temperature and Rainfall Data

This study analyses the seasonal variations in rainfall and air temperature recorded at two WWTPs, WWTP A and WWTP C, during the period of 2021–2022. The data (Table 15) include the average rainfall (mm) and air temperature (°C) for each quarter (Q4, Q1, Q2, and Q3). The monthly average temperature and precipitation data utilized in this study were provided by the National Meteorological Administration of Romania. The data were available for WWTP A and C.

### 4.4. Chemicals and Reagents

Acetonitrile of an HPLC grade was purchased from Sigma-Aldrich (Darmstadt, Germany), and formic acid was purchased from Cristal R Chim (Bucharest, Romania). For the SPE method, methanol (MeOH) was purchased from Merck (Darmstadt, Germany), the ammonium hydroxide was purchased from Primexchim (Bucharest, Romania), and the hydrochloric acid (HCl) was purchased from Poch (Gliwice, Poland). Ethylenediamine tetraacetic acid disodium salt dihydrate (EDTA) was purchased from Fluka (Buchs, Switzerland). All experiments’ high-purity water was prepared using a Mili-Q Ultrapure water purification system (Millipore, Billerica, MA, USA). Six antibiotic standards, amoxicillin (AMX), ciprofloxacin (CIP), norfloxacin (NOR), azithromycin (AZT), clarithromycin (CLT), doxycycline (DOX), were purchased from Sigma-Aldrich (Darmstadt, Germany) and piperacillin was purchased from Alpha Aesar (Kandel, Germany) (Table 16).

The stock solutions of antibiotics were obtained by dissolving 1 mg of the powder form of each substance in 1 mL of the appropriate solvent. Amoxicillin and piperacillin were dissolved in acetonitrile: aqueous formic acid 0.1% (50:50, *v*/*v*), ciprofloxacin and norfloxacin were dissolved just in aqueous formic acid 0.1%, azithromycin and clarithromycin were dissolved in acetonitrile, and doxycycline was dissolved in ultrapure water. The final stock standard solutions were stored at −20 °C. For the HPLC analysis, we used the following working standard solutions of each antibiotic: 100 µg/mL, 16 µg/mL, 14 µg/mL, 12 µg/mL, 10 µg/mL, 9 µg/mL, 8 µg/mL, 7 µg/mL, 6 µg/mL, 4 µg/mL, 2 µg/mL, 1 µg/mL, 0.8 µg/mL, and 0.6 µg/mL. Working standard solutions were obtained by diluting the stock solutions and then stored in dark containers in a refrigerator at 2–8 °C to prevent degradation.

### 4.5. Analytical Procedures

Solid-phase extraction of antibiotics from water samples was performed following a previously described method [69]. Briefly, the preconcentration of antibiotics from the water samples was carried out using Oasis HLB SPE cartridges (500 mg, 6 mL; Waters, Milford, MA, USA) on a SupelcoVisiprep SPE vacuum manifold from Sigma-Aldrich (Darmstadt, Germany). Before sample application, cartridges were conditioned with 25 mL of methanol and 25 mL of ultrapure water and then loaded with 250 mL of sample (Table 17). Prior to analysis, 0.2 g of EDTA was added to 250 mL of sample, and the pH was adjusted to 5.5 with 0.5 N of HCl or 5% NH_4_OH. The samples were passed through the cartridges at a flow rate of 2 mL/min. The antibiotics retained on the cartridges were eluted with 25 mL of methanol, the obtained methanolic solutions were evaporated to dryness using a rotary evaporator (Laborota 4011-digital; Heidolph, Schwabach, Germany) at 40 °C, and the residues were dissolved in 2 mL of ultrapure water: acetonitrile (1.25:0.75, *v*/*v*). Before analysis, the extracts were passed through nylon syringe filters (13 mm, 45 mm; Phenomenex, Torrance, CA, USA). Extractions for each sample were performed in triplicate. Antibiotics were assayed using a high-performance liquid chromatography (HPLC) system, Shimadzu 2010 (Shimadzu, Kyoto, Japan), equipped with a diode array and mass spectrometry single quadrupole (MS) detectors (Shimadzu, Kyoto, Japan). The antibiotics were separated on a Zorbax SB C18 column (100 × 3 mm, 3.5 μm) thermostated at 40 °C. The mobile phase consisted of acetonitrile: ultrapure water (90:10, *v*/*v*) (A) and 0.1% aqueous formic acid (B). The gradient program started with 5% A for 1 min, increased up to 50% A in 8 min, and then at 6 min, it reached 85% A, which was maintained for 5 min. The flow rate of the mobile phase was 0.3 mL/min and the injected sample volume was 10 μL. The mass spectrometric detection parameters were a capillary voltage 1.5 kW, a dissolution temperature of 250 °C and an interface temperature 200 °C, and positive electrospray ionization (ESI^+^). The concentrations of antibiotics in influent and effluent samples from WWTPs were determined by the standard addition method as described by Soran et al. [69,70] alongside a validation of the analytical parameters (Table 17).

### 4.6. Microbiology Assay

The bacterial load in each collected water sample was estimated using the colony forming units (CFU) technique. Nutrient agar (NA) was used as growth media for all experiments because all aerobic heterotrophs grow on this type of media. The samples were diluted ten-fold in physiological serum and 1 mL samples of the 5th to 10th dilution were transferred on plates with NA. The plates were left to incubate for 24 to 72 h at 35 °C, after which the CFU were counted via a manual method using the following formula:

$$\mathrm{colony}\;\mathrm{forming}\;\mathrm{units}\;\left(\mathrm{CFU}/\mathrm{mL}\right)=\frac{\sum \left(n\;\times \;d\right)}{N\;\times V}$$

where

n—Number of colonies in a Petri plate,
d—The inverse of the dilution of the inoculated sample,
N—The number of Petri plates considered,
V—The volume of the sample used, in mL [71].

Bacterial strains were isolated from the plates that showed CFU, performing multiple transfers until only one type of colony was developed. For the characterization of the isolated strains, the following methods were used, as outlined by Carpa et al. [71]:

1. Gram staining is a double staining that helps distinguish Gram-positive and Gram-negative bacteria from the samples. This method was used to distinguish coliforms, knowing that most of them are Gram-negative bacteria.
2. Antibiogram assay—The diffusimetric method was used, on Mueller–Hinton agar medium [72] with antibiotic disks, which is considered a method with large applicability in practice to test the efficacy of antimicrobial substances. The bacterial strain suspensions were adjusted to 0.5 McFarland turbidity, and their susceptibility for the seven antibiotics (detected through the HPLC method) was tested. Incubation was performed at 37 °C for 18–24 h; thereafter, the diameter of the inhibition zone was measured. The interpretation of the results was carried out in accordance with the EUCAST guidelines [73]. The diameter of the inhibition area is correlated with the sensitivity of the bacterium to the tested antibiotics.
3. Scanning electron microscopy (SEM) technique. The microscopic examination was performed to support the observations made by the microbiological procedures. The bacteria were taken from the plates with NA where CFU calculations were made. Then, the samples were fixed with 2.7% glutaraldehyde, washed with phosphate-buffered saline (PBS), dehydrated with 30 to 100% ethanol, and examined using a SEM Hitachi SU8230 (Hitachi, Tokyo, Japan) operated at 30 kV.

### 4.7. Calculations and Statistical Analyses

Antibiotic concentrations detected from different environments and at different dates were exported to Microsoft Excel and then analyzed in the R environment for statistical computing and graphics (R Foundation for Statistical Computing, Vienna, Austria), version 4.2.1 [74] software for statistical analysis. Continuous data were presented as median, interquartile ranges, means, and standard deviations. Comparisons between dependent observations (influent vs. effluent) were performed with the Wilcoxon rank-sum test and *t*-tests for dependent samples. Comparisons between seasons were performed with the Kruskal–Wallis test, followed by post hoc nonparametric tests. A *p*-value below 0.05 was considered to be statistically significant. For all tests, two-tailed *p*-values were computed. 

To explore the associations between antibiotic concentrations and the mean monthly temperature or the mean monthly rainfall, we built simple and multiple models with antibiotic concentrations as dependent variables, and the treatment of wastewaters and the mean monthly temperature or the mean monthly rainfall as independent variables. We verified the models’ assumptions: the normality of the residuals, the presence of heteroskedasticity, the multicollinearity, and the linearity of continuous predictors with the dependent variable. The coefficients, their confidence intervals, and *p*-values were presented for all models. The determination coefficients were reported for univariate models, while the adjusted ones were reported for multivariate models.

### 4.8. Environmental Risk Assessment (ERA)

To examine the potential impact of the antibiotics detected in the effluents from WWTPs on the aquatic ecosystem, ecological and antibiotic resistance risk assessments were performed, using the following equation [20]:

RQ = PEC/PNEC

where PEC is the “Predicted Environmental Concentration” for each antibiotic, and PNEC is the “Predicted No-Effect Concentration” (Table 18). Regarding the PNEC value used for each substance, it was either the environmental predicted no-effect concentration (PNEC-ENVs) used for the evaluation of the impact on microbial communities in aquatic systems or the PNECs based on the minimal inhibitory concentrations (PNECs-MICs) used in the assessment of the selective pressure for antibiotic resistance in microbial populations, whichever had the lower value.

The PEC was calculated using the following equation:

PEC = MC/DF

where MC is the “Measured Concentration” in the wastewater effluents for each antibiotic, and DF is the “National annual median dilution factor” calculated for each country by Keller et al. [20,75]. For Romania, the dilution factor is 71.31. Risk categorization for selected antibiotics was divided into three categories based on the RQ (risk quotient) values: low or insignificant risk to organisms (RQ ≤ 0.1), moderate risk to organisms (0.1 ≤ RQ ≤ 1), and high risk to organisms (RQ > 1).

**Table 18:** Environmental predicted no-effect concentration (PNEC-ENV) and predicted no-effect concentration based on minimum inhibitory concentration (PNEC-MIC) for the seven antibiotics selected in this study (extracted from [76]).

Antibiotic	PNEC-ENV (µg/mL)	PNEC-MIC (µg/mL)	Lowest PNEC Value (µg/mL)
**Amoxicillin**	N/A	0.016	0.016
**Piperacillin**	N/A	0.0005	0.0005
**Ciprofloxacin**	0.00045	0.00006	0.00006
**Norfloxacin**	0.0012	0.0005	0.0005
**Azithromycin**	0.00002	0.00025	0.00002
**Clarithromycin**	0.00008	0.00025	0.00008
**Doxycycline**	N/A	0.002	0.002

## 5. Conclusions

This study examined the occurrence of antibiotic residues and bacterial loads in influent and effluent samples from three WWTPs in the central-western region of Romania across four seasons. Our analysis has encompassed antibiotic removal efficiency, seasonal variations of antibiotic residues, environmental risk assessment, and the isolation and characterization of some bacterial strains potentially involved in antibiotic resistance.

The findings revealed seasonal variations in antibiotic concentrations and bacterial loads, with higher levels detected during warmer seasons. Statistically significant differences in antibiotic concentrations were observed, particularly for amoxicillin, which showed a mean difference of 7.11 µg/mL between influents and effluents (*p*-value = 0.093). The antibiotic removal efficiency varied among the WWTPs, with some antibiotics like amoxicillin being partially removed, while others like doxycycline and piperacillin persisted, especially in the autumn season, showing negative removal rates. Statistical analysis demonstrated correlations between antibiotic concentrations and environmental factors. Higher temperatures and rainfall were associated with increased concentrations of amoxicillin and decreased concentrations of doxycycline, indicating that these factors play a role in the variability of antibiotic levels in wastewater. Notably, the observed variations in pH across different seasons and treatment plants highlight the need for the careful management of pH and other physicochemical properties to enhance the overall effectiveness of antibiotic removal in wastewater treatment processes. High antibiotic concentrations in effluents pose environmental risks and potentially contribute to the development of antibiotic-resistant bacteria, with notable resistance observed for amoxicillin and clarithromycin, particularly during warmer seasons. The presence of bacterial strains was more pronounced during these warmer periods, highlighting the need for optimized treatment processes and continuous monitoring. Overall, the study underscores the importance of improving wastewater treatment methods to mitigate the environmental and public health risks associated with antibiotic residues and resistant bacteria.

## Figures and Tables

**Table 1:** Statistical analysis of antibiotic concentrations (µg/mL) in WWTP influents and effluents.

	Median		Difference (95% CI)	*p*-Value
	Influent	Effluent		
**AMX**	7.11 (<LOQ–16.61)	<LOQ (<LOQ–2.1)	7.11 (2.84–19.98)	0.093
**AZT**	0.56 (<LOQ–1.12)	<LOQ (<LOQ–1.12)	0.56 (NaN–NaN)	1
**CIP**	<LOQ (<LOQ–0.26)	0.41 (<LOQ–1.05)	−0.41 (−1.05–3.55)	0.854
**CLT**	1.27 (<LOQ–1.27)	1.27 (<LOQ–1.27)	<LOQ (−1.27–1.28)	0.584
**DOX**	2.18 (<LOQ–2.47)	<LOQ (<LOQ–2.51)	2.18 (−1.47–2.32)	0.933
**NOR**	<LOQ (<LOQ–0.79)	0.89 (0.43–1)	0.89 (−1–6.02)	0.824
**PIP**	0.87 (0.2–1.87)	0.24 (<LOQ–0.9)	0.64 (−15.73–1.79)	0.286
	**Mean**		**Difference (95% CI)**	***p*** **-Value**
	**Influent**	**Effluent**		
**AMX**	9.5 (10.47)	3.88 (9.12)	5.62 (−0.28–11.53)	0.06
**AZT**	0.56 (0.58)	0.47 (0.57)	0.09 (−0.11–0.3)	0.339
**CIP**	0.94 (2.64)	0.51 (0.53)	0.44 (−1.13–2.01)	0.553
**CLT**	0.89 (0.74)	0.84 (0.69)	0.05 (−0.31–0.41)	0.764
**DOX**	1.5 (1.36)	1.32 (1.73)	0.18 (−0.61–0.97)	0.622
**NOR**	2.23 (5.09)	1.05 (1.13)	1.19 (−2.11–4.48)	0.445
**PIP**	1.21 (1.18)	3.26 (10.02)	−2.05 (−8.53–4.43)	0.501

AMX—amoxicillin, AZT—azithromycin, CIP—ciprofloxacin, CLT—clarithromycin, DOX—doxycycline, NOR—norfloxacin, PIP—piperacillin.

**Table 2:** Antibiotic concentrations in WWTP influents (I) and effluents (E): a statistical descriptive analysis by season (µg/mL).

	Median				*p*-Value
	Q4 (n = 3)	Q1 (n = 3)	Q2 (n = 3)	Q3 (n = 3)	*p* {(1, 2)/(1, 3)/(1, 4)/(2, 3)/(2, 4)/(3, 4)}
**AMX I**	n.d.	n.d.	14.65 (14.43–19.99)	19.98 (17.74–22.16)	0.005 {1/<0.001/<0.001/<0.001/<0.001/0.998}
**AMX E**	n.d.	n.d.	10.47 (6.79–20.82)	<LOQ (<LOQ–0.88)	0.018 {1/<0.001/0.889/<0.001/0.889/<0.001}
**AZT I**	1.12 (1.12–1.12)	n.d.	1.12 (1.12–1.12)	n.d.	0.005 {<0.001/1/<0.001/<0.001/1/<0.001}
**AZT E**	1.12 (1.12–1.12)	n.d.	1.12 (0.56–1.12)	n.d.	0.044 {<0.001/0.827/<0.001/0.548/1/0.548}
**CIP I**	n.d.	n.d.	1.05 (1.05–5.13)	n.d.	0.018 {1/<0.001/1/<0.001/1/<0.001}
**CIP E**	1.05 (0.93–1.05)	n.d.	1.05 (1.05–1.05)	n.d.	0.007 {<0.001/0.928/<0.001/<0.001/1/<0.001}
**CLT I**	1.27 (1.27–1.27)	1.27 (0.9–1.54)	1.27 (0.64–1.63)	n.d.	0.108 {1/1/<0.001/1/<0.001/0.594}
**CLT E**	1.27 (1.27–1.27)	0.52 (0.26–0.89)	1.27 (1.27–1.6)	n.d.	0.017 {0.629/0.886/<0.001/0.408/0.629/<0.001}
**DOX I**	2.2 (2.18–2.72)	2.74 (2.56–2.89)	n.d.	<LOQ (<LOQ–1.13)	0.025 {0.995/<0.001/0.916/<0.001/<0.001/0.895}
**DOX E**	3.9 (3.38–4.17)	2.23 (1.11–2.31)	n.d.	n.d.	0.004 {<0.001/<0.001/<0.001/0.629/0.629/1}
**NOR I**	n.d.	n.d.	8.19 (4.1–12.38)	0.55 (0.28–1.02)	0.107 {1/0.459/0.459/0.459/0.459/0.878}
**NOR E**	1 (0.89–1)	1 (0.93–2.48)	0.92 (0.75–1.7)	n.d.	0.049 {0.984/1/<0.001/0.988/<0.001/<0.001}
**PIP I**	1.74 (1.29–2.62)	1.69 (0.85–1.98)	0.9 (0.58–1.82)	<LOQ (<LOQ–0.28)	0.228 {0.983/0.983/<0.001/1/0.865/0.638}
**PIP E**	0.9 (0.9–17.98)	n.d.	<LOQ (<LOQ–0.45)	0.45 (0.24–0.68)	0.024 {<0.001/0.37/0.37/0.853/<0.001/0.951}

n.d. = not detected, AMX—amoxicillin, AZT—azithromycin, CIP—ciprofloxacin, CLT—clarithromycin, DOX—doxycycline, NOR—norfloxacin, PIP—piperacillin, I—influent, E—effluent.

**Table 3:** Antibiotic removal efficiency (RE %) from three WWTPs during four seasons.

Location	AMX	PIP	CIP	NOR	AZT	CLT	DOX
**WWTP A Q4**	n/a	74%	n/a	n/a	n/a	n/a	−30%
**WWTP B Q4**	n/a	−315%	n/a	n/a	n/a	n/a	−20%
**WWTP C Q4**	n/a	48%	n/a	n/a	n/a	n/a	−105%
**WWTP A Q1**	n/a	100%	n/a	n/a	n/a	100%	21%
**WWTP B Q1**	n/a	100%	n/a	n/a	n/a	71%	100%
**WWTP C Q1**	n/a	n/a	n/a	n/a	n/a	n/a	19%
**WWTP A Q2**	26%	−237%	n/a	n/a	n/a	3%	n/a
**WWTP B Q2**	78%	100%	89%	97%	n/a	n/a	n/a
**WWTP C Q2**	−23%	n/a	n/a	70%	n/a	n/a	n/a
**WWTP A Q3**	100%	n/a	n/a	n/a	n/a	n/a	100%
**WWTP B Q3**	100%	96%	n/a	100%	n/a	n/a	n/a
**WWTP C Q3**	93%	n/a	n/a	100%	n/a	n/a	n/a

AMX—amoxicillin, AZT—azithromycin, CIP—ciprofloxacin, CLT—clarithromycin, DOX—doxycycline, NOR—norfloxacin, PIP—piperacillin, WWTP: Wastewater treatment plant, Q1—Winter, Q2—Spring, Q3—Summer, Q4—Autumn, n/a—not applicable.

**Table 4:** Statistical data for the antibiotic removal efficiency.

Antibiotics	Mean (SD)/Median (IQR)
**AMX**	85.74 (39.45–98.19)
**AZT**	0 (0–0)
**CLT**	0 (0–20.34)
**DOX**	18.69 (−25.38–60.72)
**NOR**	98.27 (89.87–100)
**PIP**	95.54 (48.11–100)

AMX—amoxicillin, AZT—azithromycin, CLT—clarithromycin, DOX—doxycycline, NOR—norfloxacin, PIP—piperacillin.

**Table 5:** Logarithm of CFU/mL across the four seasons during which the samples were collected.

	WWTP A		WWTP B		WWTP C	
	Influent	Effluent	Influent	Effluent	Influent	Effluent
**Autumn Q4**	6.903	6.322	6.114	6.301	6.041	6.041
**Winter Q1**	-	6	6.477	-	7.491	-
**Spring Q2**	8.857	6.699	9.230	-	8.146	7.681
**Summer Q3**	7.633	-	8.447	-	6	-

**Table 6:** Gram staining and distribution of the eight bacterial isolates.

	WWTP A		WWTP B		WWTP C	
	Influent	Effluent	Influent	Effluent	Influent	Effluent
**Autumn Q4**	-	-	-	-	-	-
**Winter Q1**	-	-	-	-	-	-
**Spring Q2**	G+	G−	G−	-	G−	G−
**Summer Q3**	-	-	G−	-	-	-

G+ = Gram-positive, G− = Gram-negative, “-” inconclusive isolates.

**Table 7:** Simple and multiple linear regression models predicting amoxicillin levels based on treatment and mean monthly temperature.

Variables	B Unadjusted	(95% CI)	*p*	R^2^	B Adjusted	(95% CI)	*p*
Intercept					1.66	(–7.65–10.96)	0.707
Treated	–5.62	(−13.94–2.69)	0.175	0.08	−4.5	(–14.27–5.28)	0.338
Mean monthly temperature (°C)	0.79	(0.2–1.37)	0.012	0.37	0.79	(0.19–1.38)	0.003

CI, confidence interval; R^2^, determination coefficient. The multivariate model included both variables. The adjusted determination coefficient was 0.32.

**Table 8:** Simple and multiple linear regression models predicting amoxicillin levels based on treatment and mean monthly rainfall.

Variables	B Unadjusted	(95% CI)	*p*	R^2^	B Adjusted	(95% CI)	*p*
Intercept					2.11	−5.07	0.574
Treated	−5.62	(−3.48–2.23)	0.175	0.08	−4.5	(−4.58–5.59)	0.398
Mean monthly rainfall (L/m^2^)	0.15	(0.06–0.24)	0.006	0.2	0.15	(0.07–0.23)	0.003

CI, confidence interval; R^2^, determination coefficient. The multivariate model included both variables. The adjusted determination coefficient was 0.13.

**Table 9:** Simple and multiple linear regression models predicting doxycycline levels based on treatment and mean monthly temperature.

Variables	B Unadjusted	(95% CI)	*p*-Value	R^2^	B Adjusted	(95% CI)	*p*
Intercept					2.99	(1.98–4)	<0.001
Treated	−0.18	(−1.5–1.14)	0.777	0.004	−0.06	(−1.12–1.01)	0.907
Mean monthly temperature (°C)	−0.14	(−0.2–−0.08)	<0.001	0.62	−0.14	(−0.2–−0.07)	<0.001

CI, confidence interval; R^2^, determination coefficient. The multivariate model included both variables. The adjusted determination coefficient was 0.56.

**Table 10:** Simple and multiple linear regression models predicting doxycycline levels based on treatment and mean monthly rainfall.

Variables	B Unadjusted	(95% CI)	*p*	R^2^	B Adjusted	(95% CI)	*p*
Intercept					3.11	(1.7–4.51)	<0.001
Treated	−0.18	(−1.5–1.14)	0.777	0.004	−0.06	(−1.35–1.24)	0.924
Mean monthly rainfall (L/m^2^)	−0.03	(−0.05–−0.01)	0.006	0.43	−0.03	(−0.05–−0.01)	0.008

CI, confidence interval; R^2^, determination coefficient. The multivariate model included both variables. The adjusted determination coefficient was 0.35.

**Table 11:** Risk quotients calculated for the antibiotics in the effluents of three WWTPs.

Effluent		AMX	PIP	CIP	NOR	AZT	CLT	DOX
**WWTP A**	Q1	0	0	0	111.1	0	0	16.74
	Q2	9.17	0	0	216.03	0	337.14	0
	Q3	0	12.68	0	0	0	0	0
	Q4	0	0	0	0	0	0	20.1
**WWTP B**	Q1	0	0	0	0	0	91.08	0
	Q2	2.73	0	0	16.07	0	0	0
	Q3	0	0.7	0	0	0	0	0
	Q4	0	98.32	0	0	0	0	27.35
**WWTP C**	Q1	0	0	0	23.97	0	0	0.02
	Q2	27.33	0	0	69.29	0	0	0
	Q3	1.54	0	0	0	0	0	0
	Q4	0	0	190.2	22.13	0	0	27.35

AMX—amoxicillin, AZT—azithromycin, CIP—ciprofloxacin, CLT—clarithromycin, DOX—doxycycline, NOR—norfloxacin, PIP—piperacillin, WWTP: Wastewater treatment plant, Q1—Winter, Q2—Spring, Q3—Summer, Q4—Autumn.

**Table 12:** Antibiograms of the eight isolated bacteria incubated with the seven most abundant antibiotics.

Location	AMX	PIP	CIP	NOR	CLT	AZT	DOX
**Q4 EWWTP C**	*IR*	S	S	S	S	S	S
**Q4 EWWTP A**	S	S	S	S	S	S	S
**Q2 E** **WWTP A**	R	S	S	S	R	R	S
**Q2 IWWTP A**	S	S	R	R	S	S	*IR*
**Q2 IWWTP C**	R	R	S	S	S	S	S
**Q2 EWWTP C**	R	*IR*	*IR*	*IR*	R	*IR*	*IR*
**Q2 IWWTP B**	S	S	S	S	R	S	S
**Q3 IWWTP B**	R	S	S	S	R	S	S

**R** = resistant, *IR* = intermediate resistance, S = sensitive, AMX—amoxicillin, AZT—azithromycin, CIP—ciprofloxacin, CLT—clarithromycin, DOX—doxycycline, NOR—norfloxacin, PIP—piperacillin, WWTP: Wastewater treatment plant, Q1—Winter, Q2—Spring, Q3—Summer, Q4—Autumn, I—influent, E—effluent.

**Table 13:** Comparative description of treatment stages in selected wastewater treatment plants.

WWTP	Mechanical Pre-Treatment	Biological Treatment	Sludge Treatment	Tertiary Treatment
WWTP A	Screening, sand and grease removal, primary sedimentation	Activated sludge process, biological reactors	Anaerobic digesters, sludge thickening, dewatering	Tertiary filtration, disinfection
WWTP B	Coarse screening, fine screening, grit chamber, primary sedimentation	Aeration tanks	Sludge thickening, dewatering, stabilization	Chemical phosphorus removal, disinfection
WWTP C	Mechanical bar screen, grit chamber, primary sedimentation	Aeration tanks	Sludge thickening, anaerobic digestion, dewatering	Tertiary filtration, UV disinfection

WWTP: Wastewater treatment plant.

**Table 14:** Seasonal pH values of influents and effluents in three WWTPs.

Season	WWTP A		WWTP B		WWTP C	
	Influent	Effluent	Influent	Effluent	Influent	Effluent
**Q1**	7.34	7.09	6.97	6.73	7.47	7.37
**Q2**	6.76	7.05	7.11	6.93	6.86	7.59
**Q3**	6.88	7.08	6.58	6.52	6.45	5.86
**Q4**	7.29	7.18	6.2	6.36	6.96	7.26

WWTP: Wastewater treatment plant, Q1—Winter, Q2—Spring, Q3—Summer, Q4—Autumn.

**Table 15:** Seasonal rainfall (mm) and air temperature (°C) at WWTPs A and C.

	Season	Recording Month	Rainfall (R24) *	Air Temperature (°C) *
**WWTP A**	Q4	November	23	4.2
	Q1	February	11.4	1.8
	Q2	May	97.4	15.4
	Q3	August	87.8	21.3
**WWTP C**	Q4	November	51.4	3.6
	Q1	February	13.8	0.8
	Q2	May	52.6	15.5
	Q3	August	77.6	21.6

WWTP: Wastewater treatment plant, Q1—Winter, Q2—Spring, Q3—Summer, Q4—Autumn, * Monthly average.

**Table 16:** Characteristics of the antibiotics studied.

Chemical Group	Antibiotic	Use	Chemical Formula	Molecular Weight (g mol^−1^)
**β-lactams**	Amoxicillin	Human and Veterinary	C_16_H_19_N_3_O_5_S	365.4
	Piperacillin	Human	C_23_H_27_N_5_O_7_S	516.54
**Fluoroquinolones**	Ciprofloxacin	Human and Veterinary	C₁₇H₁₈FN₃O₃	331.34
	Norfloxacin	Human	C_16_H_18_FN_3_O_3_	319.33
**Macrolides**	Azithromycin	Human	C_38_H_72_N_2_O_12_	748.99
	Clarithromycin	Human	C_38_H_69_NO_13_	747.95
**Tetracyclines**	Doxycycline	Human and Veterinary	C_22_H_24_N_2_O_8_·H_2_O	444.4

**Table 17:** Analytical method validation parameters.

Compound	Linearity (R^2^)	LOD µg/mL	LOQ µg/mL	Retention Time (min)	Molecular Ion (*m*/*z*)
**Amoxicillin**	0.9989	3.139	4.240	5.5	366 [M + H]^+^
**Piperacillin**	0.9993	0.908	1.804	13.75	518 [M + H]^+^
**Ciprofloxacin**	0.999	1.060	2.104	10.8	332 [M + H]^+^
**Norfloxacin**	0.9991	1.008	2.001	11.3	320 [M + H]^+^
**Azithromycin**	0.9989	1.125	2.232	12.5	749 [M + H]^+^ 375 [M + 2H]^+^
**Clarithromycin**	0.9986	1.282	2.540	15.5	748 [M + H]^+^
**Doxycycline**	0.9987	4.230	5.445	13	445 [M + H]^+^

LOD—Limit of detection, LOQ—Limit of quantification.

## Data Availability

The data presented in this study are available from the corresponding authors (S.I.P. and B.K.) upon reasonable request.
